# Association of Fenofibrate and Diabetic Retinopathy in Type 2 Diabetic Patients: A Population-Based Retrospective Cohort Study in Taiwan

**DOI:** 10.3390/medicina56080385

**Published:** 2020-07-31

**Authors:** Ying-Chieh Lin, Yu-Ching Chen, Jorng-Tzong Horng, Jui-Ming Chen

**Affiliations:** 1 Department of Bioinformatics and Medical Engineering, Asia University, Taichung 41354, Taiwan; yinchichlin@gmail.com (Y.-C.L.); yuching33@gmail.com (Y.-C.C.); horng@db.csie.ncu.edu.tw (J.-T.H.); 2 Department of Computer Science and Information Engineering, National Central University, Chungli 32001, Taiwan; 3 Department of Endocrinology and Metabolism, Tungs’ Taichung MetroHarbor Hospital, Taichung 43503, Taiwan

**Keywords:** fenofibrate, diabetic retinopathy, type 2 diabetic

## Abstract

*Background and Objectives:* Fenofibrate, a PPAR-α agonist, has been demonstrated to reduce the progression of diabetic retinopathy (DR) and the need for laser treatment in a FIELD (Fenofibrate Intervention and Event Lowering in Diabetes) study. However, in the subgroup of patients without pre-existing DR, there was no significant difference in the progression of DR between the fenofibrate group and the placebo group. In this study, we aim to investigate whether fenofibrate can decrease the risk of incident DR in a population-based cohort study of type 2 diabetic patients in Taiwan. *Materials and Methods:* A total of 32,253 type 2 diabetic patients without previous retinopathy were retrieved from 892,419 patients in 2001–2002. They were then divided into two groups based on whether they were exposed to fenofibrate or not. The patients were followed until a diagnosis of diabetic retinopathy was made or until the year 2008. *Results:* With a follow-up period of 6.8 ± 1.5 years and 5.4 ± 2.6 years for 2500 fenofibrate users and 29,753 non-users, respectively, the Cox proportional hazard regression analysis revealed that the hazard ratio (HR) of new onset retinopathy was 0.57 (95% CI 0.57–0.62, *p* < 0.001). After adjusting for hypertension; the Charlson comorbidity index (CCI); and medications such as angiotensin-converting enzyme inhibitors (ACE-I), angiotensin receptor blockers (ARB), anticoagulants, gemfibrozil, statins, and hypoglycemic agents, the adjusted HR was 0.75 (95% CI 0.68–0.82, *p* < 0.001). The need for laser treatment has an HR and adjusted HR of 0.59 (95% CI 0.49–0.71, *p* < 0.001) and 0.67 (95% CI 0.56–0.81, *p* < 0.001), respectively. *Conclusion:* Our study showed that the long-term and regular use of fenofibrate may decrease the risk of incident retinopathy and the need for laser treatment in type 2 diabetic patients. Since there are limitations associated with our study, further investigations are necessary to confirm such an association.

## 1. Introduction

The association of fenofibrate and diabetic retinopathy (DR) has been studied extensively due to the high prevalence of DR in diabetic patients. A recent systematic review of 35 population-based studies showed that the prevalence of DR, proliferative diabetic retinopathy (PDR), diabetic macular edema (DME), and vision threatening DR (VTDR) among individuals with diabetes was 34.6%, 7.0%, 6.8%, and 10.2%, respectively [1]. Another study in rural China projected that 21.1 million people aged 30 years and above have diabetes and 9.2 million have DR, including 1.3 million with vision-threatening DR (VTDR) [2]. In developed countries, diabetic retinopathy is the leading cause of visual loss in adults of working age (20–65 years) [3]. 

Although intensive glycemic control has been shown to reduce diabetic retinopathy [4,5], there is, however, a two- to three-fold increase in the risk of severe hypoglycemia [5]. In the past years, novel approaches to the medical treatment of diabetic retinopathy have emerged. Drugs that act on the renin-angiotensin system (RAS), including angiotensin-converting enzyme inhibitors (ACE-I, e.g., lisinopril and perindopril) and angiotensin receptor blockers (ARB, e.g., candesartan), are reported to have beneficial effects on DR [6]. Fenofibrate, a PPAR-α (peroxisome proliferator-activated receptor alpha) agonist, is usually prescribed for lowering mainly triglycerides and was demonstrated to reduce the progression of DR and the need for laser treatment in the FIELD (Fenofibrate Intervention and Event Lowering in Diabetes) study [7]. However, the DR endpoint was only a tertiary objective. Moreover, in the subgroup of patients without pre-existing diabetic retinopathy, the FIELD study did not show any difference in the progression of DR in the fenofibrate group as compared to the placebo group. The Accord-Eye study, using fenofibrate combined with statin, also demonstrated a reduced progression of DR [8]. On the other hand, the Collaborative Atorvastatin Diabetes Study (CARDS) study, which showed the effectiveness of statins (atorvastatin) in the primary prevention of cardiovascular disease in diabetic patients, did not show an effect on DR progression [9]. Since there has been little focus on the effectiveness of fenofibrate in preventing retinopathy in type 2 diabetic patients, we used the data from Taiwan’s National Health Insurance Research Database (NHIRD) [10] to elucidate the issue of whether fenofibrate can be effective in the primary prevention of DR and decrease the need for laser treatment in type 2 diabetic patients.

## 2. Methods

### 2.1. Data Sources

The national health insurance program of Taiwan was implemented in 1995, and 95% of residents were enrolled. The insurance coverage rates in 2007 and 2014 were 98.4% and 99%, respectively [10]. Data used in this research contained 1 million subjects randomly selected from the NHIRD in 1999–2008; the database contained disease information of inpatients and outpatients, prescription drugs, dates of treatments, gender, birth date, and other relevant information. Program data also contained registration files and original claim data for reimbursement. Large computerized de-identified databases derived from this system by the Bureau of National Health Insurance, Taiwan (BNHI) and maintained by the National Health Research Institutes, Taiwan are provided to scientists in Taiwan for research purposes. Data in the National Health Insurance Research Database (NHIRD) that could be used to identify patients or care providers, including medical institutions and physicians, are scrambled before being sent to the National Health Research Institutes for database construction and are further scrambled before being released to each researcher. Theoretically, it is impossible to query the data alone to identify individuals at any level using this database [10]. As the data files consisted of unidentified secondary data, the study was exempted from a full review by the Institutional Review Board of Tungs’ Taichung MetroHarbor Hospital (IRB TTMHH) in 2014. Obtaining informed consent from the study population was not required due to the de-identified data files, the large size of the population, and the deceased status of some of the population by the time of the study (IRB TTMHH No.:103204 N).

### 2.2. Subject Selection

There was a total of 892,419 patients from one million NHIRD individuals in 2001–2002. Patients who had at least three outpatient visits or at least one hospitalization with a recorded diagnosis using the International Classification of Diseases, Ninth Revision, Clinical Modification (ICD-9-CM) code for DM (250.xx) were included in this study.

**Figure 1:**
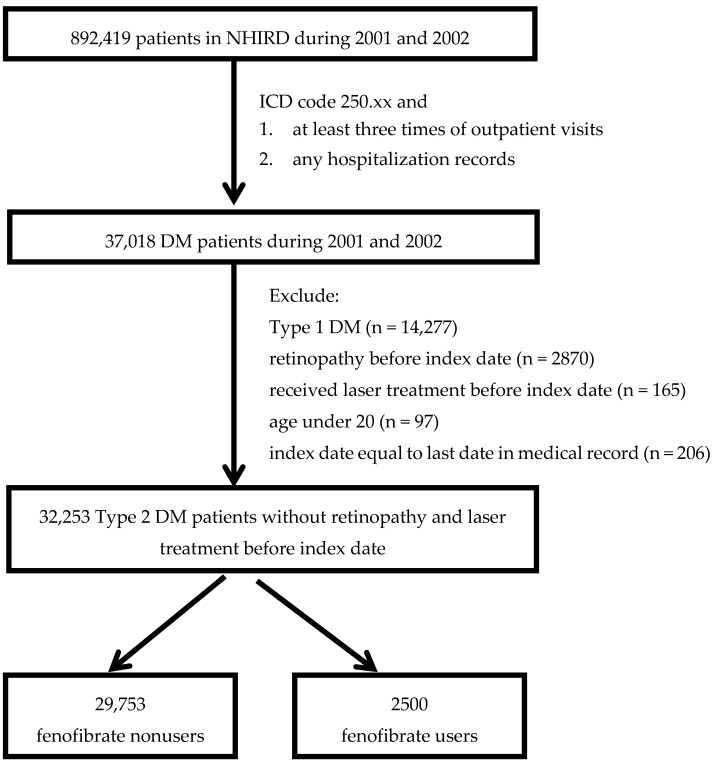
Study subjects selection flow chart. Abbreviations: NHIRD: Taiwan’s National Health Insurance Research Database, DM: diabetes mellitus, ICD code: International Classification of Diseases.

After excluding patients with type 1 diabetes mellitus (T1DM, ICD-9-CM codes 250.x1 and 250.x3), pre-existing retinopathy (ICD-9-CM codes 250.5x and 361.8 to 362.9), previous laser treatment (visit codes for pan-retinal photocoagulation, PRP, were 60003C and 60004C and, for focal laser of peripheral retina, were 60004C and 60005C) before the index date, and age under 20 years, 32,253 type 2 diabetic patients without pre-existing retinopathy and prior laser treatment before the index date in 2001–2002 were recruited (Figure 1).

These patients were then divided into two groups according to fenofibrate use or non-use and were followed up until 2008. The primary outcome was defined as newly diagnosed diabetic retinopathy or the receipt of laser treatment. The defined daily dose (DDD) of fenofibrate in the ATC (Anatomical Therapeutic Chemical)/DDD system is 200 mg. Patients whose cumulative duration of treatment of fenofibrate was under 3 months (<18,000 mg) during the study period were defined as non-fenofibrate users (n = 29,753); otherwise, they were treated as fenofibrate users (n = 2500). Cumulative durations of treatment with fenofibrate were further stratified into 0 to 3 months (0–3 M), 3 months to 1 year (3 M–1 y), 1 year to 2 years (1–2 y), and longer than 2 years (>2 y).

The risk factors and confounders of diabetic retinopathy were also considered. Hazard ratios (HRs) were adjusted for hypertension (ICD-9-CM code 401 to 405); the Charlson comorbidity index (CCI) [11,12]; and medications such as angiotensin-converting enzyme (ACE) inhibitors (ACE-I), angiotensin receptor blockers (ARB), anticoagulants, gemfibrozil, statins, and hypoglycemic agents. 

ACE-I included benazepril, cilazapril, enalapril, imidapril, lisinopril, perindopril, quinapril, ramipril, fosinopril, and captopril. ARB included candesartan, irbesartan, losartan, olmesartan, telmisartan, and valsartan. Anticoagulants included aspirin, dipyridamole, and ticlopidine. Statins included for analysis were atorvastatin, fluvastatin, lovastatin, pravastatin, rosuvastatin, and simvastatin. Hypoglycemic agents were grouped as metformin; thiazolidinedione (TZD, rosiglitazone, and pioglitazone); sulfonylureas (glipizide, gliclazide, and glimepiride); glinide (repaglinide and nateglinide); acarbose; and insulin.

The index date was the first date to retrieve patients with type 2 diabetes in 2001–2002, and the end date was the date of retinopathy diagnosis, the date of death, or the date of the last record in the NHIRD, whichever came first. 

### 2.3. Statistical Analysis

The differences between the fenofibrate group and non-fenofibrate group in type 2 diabetes mellitus (T2DM) patients were compared using the chi-square test and Student’s *t*-test for categorical and continuous variables, respectively. The hazard function of the Cox regression model with time-varying covariates is $h\left(t,X\right)=h_{0}\left(t\right)\exp(\overset{p}{\underset{i=1}{\sum }}\left[\beta_{i}X_{i}+\delta_{i}X_{i}\times g_{i}\left(t\right)\right])$, where $h_{0}\left(t\right)$ represents the baseline hazard, $X=\left(X_{1},X_{2},\ldots ,X_{p}\right)$ represents a collection of *p* predictor variables, and $g_{i}\left(t\right)$ is used to define the time function for the ith predictor. Predictors such as fenofibrate, age, gender, hypertension, CCI, ACE inhibitors, ARBs, anticoagulant drugs, gemfibrozil, statins, and hypoglycemic agents were considered in this study. When the product term is removed from the Cox regression model, the formula is reduced to $h\left(t,X\right)=h_{0}\left(t\right)\exp(\overset{p}{\underset{i=1}{\sum }}\beta_{i}X_{i})$ and is known as the Cox proportional hazard (PH) model. The PH model assumes that the hazard is constant over time or the hazard for one individual is proportional to the hazard for another individual, where the proportionality constant is independent of time. This also means that the hazard ratio (HR) should be a constant in the Cox PH model. On the other hand, the hazard changes with time in the Cox regression model, so its HR also varies with time. In this study, time-dependent variables were used to assess the PH assumption for time-independent variables by testing the significance of the product term in the Cox regression model. Only gender, simvastatin, and insulin did not violate the PH assumption; therefore, the Cox regression model with time-varying covariates was used to estimate the hazard ratio with 95% CI in this study. The model can be used to evaluate the association between fenofibrate and the events of retinopathy or laser treatment. IBM (Armonk, NY, USA) SPSS Statistics 22 was utilized for statistical computation in this study.

## 3. Results

### Demographic Characteristics

The study population included 32,253 T2DM patients whose ages were greater than 20 years old in 2001–2002 and did not have a history of retinopathy or laser treatment before recruitment. In this study population, 29,753 subjects were defined as non-users of fenofibrate, and 2500 patients were regular fenofibrate users (Table 1).

The mean ± SD ages of these two groups at the time of T2DM diagnosis were 60.7 ± 12.8 years and 56.4 ± 12.0 years, respectively. The fenofibrate users were significantly younger than non-users (*p* < 0.001). Males comprised 54.3% and 50.0% in the fenofibrate user group and non-user group, respectively; significantly more males were in the fenofibrate user group (*p* < 0.001). The median (interquartile range, IQR) follow-up years for patients receiving fenofibrate was 7.5 (1.5); this was significantly longer than that of non-users, which was 6.5 (4.6). Furthermore, fenofibrate users had more hypertension history (83.6%) than patients not using fenofibrate (75.1%). The mean ± SD of the CCI for non-users and fenofibrate users was similar at 4.8 ± 2.9 and 4.8 ± 2.7, respectively (*p* = 0.195). However, the distributions of the CCI in both groups were not similar (*p* < 0.001).

More medications, including angiotensin-converting enzyme (ACE) inhibitors, angiotensin receptor blockers (ARB), anticoagulation agents, gemfibrozil, statins, and oral hypoglycemic agents, were prescribed in the group of fenofibrate users. Only insulin had a higher proportion in the non-user group (0.4%) than in the fenofibrate user group (0.2%), but this was not statistically significant (*p* = 0.083).

**Figure 2:**
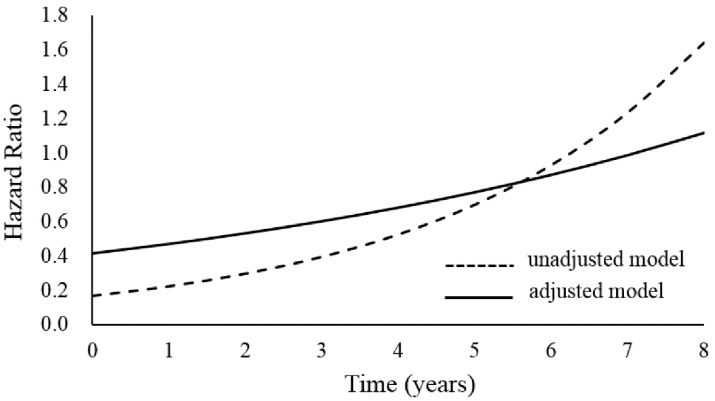
Survival curves of retinopathy.

At the end of the follow-up, the incidence of retinopathy was higher in patients not using fenofibrate than in patients using fenofibrate (27.48% vs. 19.68%, Table 2). The Cox regression analysis with time in the fenofibrate status revealed that the retinopathy HR increased from 0.17 (95% CI 0.13–0.21) to 1.64 (95% CI 1.39–1.95), and the adjusted retinopathy HR ranged from 0.42 (95% CI 0.33–0.53) to 1.12 (95% CI 0.93–1.33) within eight years. Age, gender, hypertension, CCI, ACE inhibitor, ARB, anticoagulant agents, gemfibrozil, statins, and hypoglycemic agents were adjusted in this study. According to Figure 2, at time 0, diabetic patients receiving fenofibrate were 0.17 and 0.42-times more likely to develop retinopathy compared to those not receiving fenofibrate in unadjusted and adjusted models, respectively. The dashed line increases sharply after the fourth year, while the solid line steadily increases throughout the whole duration. Furthermore, the adjusted HRs were lower than one, which was significant in the initial six years; it also indicated that patients receiving fenofibrate might have less a chance of developing retinopathy compared to patients not receiving fenofibrate. However, the adjusted HR was larger than one, which was not statistically significant in the last year. Moreover, patients were further stratified into four sub-cohorts based on their cumulative duration treatment of fenofibrate for analysis. In Supplemental Table S1, the percentages of retinopathy events were 27.48%, 21.20%, 17.14%, and 15.13% in the groups with 0–3 M, 3 M–1 y, 1–2 y, and > 2 y cumulative duration treatment, respectively. The adjusted HR was 0.52 (95% CI 0.40–0.68) to 1.12 (95% CI 0.91–1.38), 0.28 (95% CI 0.15–0.51) to 1.16 (95% CI 0.80–1.67), and 0.07 (95% CI 0.02–0.22) to 1.44 (95% CI 0.86–2.39) for the cohorts in 3 M–1 y, 1 y to 2 y, and >2 y, respectively. The adjusted retinopathy hazard ratios for the T2 DM cohorts from 2001 to 2008 are presented in Supplemental Figure S1, and the hazard rate of the group 0–3 M cumulative duration treatment is designated as the reference. There was an increased HR in each cumulative duration treatment, and the HRs increased to one in the final year, but this was insignificant. Furthermore, longer cumulative duration treatments had smaller HRs in the first six years, although their HRs were similar in the seventh year. Finally, the longest cumulative duration treatment (>2 y) had a higher HR than the others in the final year.

Supplemental Table S2 shows that the incidence of laser treatments was higher in the non-user group (6.46%) than the fenofibrate user group (4.88%). The laser treatment HR was 0.15 (95% CI 0.09–0.25) to 1.64 (95% CI 1.18–2.28) and 0.31 (95% CI 0.18–0.52) to 1.10 (95% CI 0.78–1.54) in the unadjusted model and adjusted model, respectively. The laser treatment hazard ratio curves for the T2 DM cohort from 2001 to 2008 are shown in Supplemental Figure S1 and demonstrate that the adjusted HR increased slightly compared to the unadjusted HR. Although the adjusted HR was above one in the final year, it was not significant.

## 4. Discussion

In this study, we were able to demonstrate that a long-term use of fenofibrate can result in a decreased incidence of retinopathy in type 2 diabetic patients, and the effect was dose-dependent. Our study also showed a decrease in the need for laser treatments in the study cohort.

In the FIELD (Fenofibrate Intervention and Event Lowering in Diabetes) study, 4895 diabetic patients treated with fenofibrate at a daily dose of 200 mg showed a reduction in the need for laser treatment by 31% after five years of follow up [7]. However, in the ophthalmology sub-study of 488 patients without pre-existing retinopathy, no difference was found in the progression of DR between patients treated with fenofibrate and those without exposure to fenofibrate (43 (11.4%) vs. 43 (11.7%), *p* = 0.87) after five years of follow up. This result was not corroborated by our study. In our study, the incidence of newly diagnosed retinopathy decreased after the regular use of fenofibrate and was dose-dependent after about six point eight years of follow up. The major difference between our study and the FIELD ophthalmology sub-study was that our study cohort was larger in scale (2500 vs. 488), with a slightly longer follow-up period. Another difference was the basis for the diagnosis of retinopathy. The diagnosis of diabetic retinopathy in our study was based on the ICD-9 code and not on routine retinal photography, as was the case in the FIELD ophthalmology sub-study. Moreover, the FIELD study was a multinational randomized trial, while ours was a population-based study consisting of only one ethnic group.

In our study cohort, the proportion of patients with hypertension was higher in fenofibrate users than in non-users. As a result, the fenofibrate user group was prescribed with more ACE-I and ARB. There were some previous investigations that reported the benefits of ACE-I and ARB on diabetic retinopathy [6,13,14].

The definite mechanism of fenofibrate in decreasing the progression of diabetic retinopathy is still not fully understood. Many possible lipid-related or non-lipid-related mechanisms have been postulated. Villarroel et al. reported that the ability of fenofibrate to suppress AMPK (AMP-activated protein kinase) may prevent the disruption of retinal pigment epithelium by interleukin (IL)-1β [15]. Kim et al. also proposed that fenofibrate can prevent apoptotic cell death through the AMPK-dependent pathway [16]. Fenofibrate may also downregulate basement membrane components to protect against outer blood-retinal-barrier leakage associated with diabetic retinopathy [17].

Ying Chen et al. reported that fenofibrate may offer benefits for diabetic macular edema and diabetic retinopathy via direct effects on retinal inflammation, retinal vascular leakage, and retinal neovascularization [18].

Our study utilized a large-scale population-based cohort with a relatively longer follow-up period in assessing the effects of fenofibrate on diabetic retinopathy prevention. As a retrospective cohort study based on a nationwide claim database, there are some limitations in our study. First, the diagnosis of retinopathy was based on ICD codes and not routine retinal photography. The ICD code may not fully reflect and accurately diagnose retinopathy. Diabetic patients in Taiwan may not routinely receive a screening of retinal photography, and this could result in a low detection rate of retinopathy. Our claim data also cannot provide profiles about glycemic, lipid, and blood-pressure control, although in the FIELD ophthalmology sub-study and other studies, serum lipids were deemed unrelated to the progression of diabetic retinopathy or the development of proliferative diabetic retinopathy [7,17,19]. On the other hand, the Accord-Eye study demonstrated that intensive glycemic control combined with a lipid-lowering treatment, but not intensive blood-pressure control, reduced the progression of diabetic retinopathy [8].

## 5. Conclusions

Our study showed that the long-term and regular use of fenofibrate may decrease the risk of developing retinopathy and the need for laser treatment in type 2 diabetic patients. Since there are limitations in our study, further investigations are necessary to confirm the association between fenofibrate and incident retinopathy in type 2 diabetic patients.

## Figures and Tables

**Table 1:** Demographics.

Descriptor	No Fenofibrate		Fenofibrate		
Cases	29,753	(%)	2500	(%)	*p*-Value
**Age group\Mean ± SD**	60.7 ± 12.8		56.4 ± 12.0		<0.001
20–29	289	(1.0)	19	(0.8)	<0.001
30–39	1269	(4.3)	175	(7.0)	
40–49	4579	(15.4)	592	(23.7)	
50–59	7152	(24.0)	682	(27.3)	
60–69	8355	(28.1)	640	(25.6)	
70–79	6456	(21.7)	341	(13.6)	
≧80	1653	(5.6)	51	(2.0)	
**Gender**					
Male	14,873	(50.0)	1358	(54.3)	<0.001
Female	14,880	(50.0)	1142	(45.7)	
**Follow up group\Median (IQR)**	6.5(4.6)		7.5 (1.5)		
y≤ 3	7055	(23.7)	101	(4.0)	<0.001
3<y≤5	3843	(12.9)	220	(8.8)	
5<y≤7	6322	(21.2)	645	(25.8)	
y>7	12,533	(42.1)	1534	(61.4)	
**Chronic diseases**					
Hypertension	22,339	(75.1)	2089	(83.6)	<0.001
**CCI group\Mean ± SD**	4.8 ± 2.9			4.8 ± 2.7	0.195
CCI score 1	2923	(9.8)	197	(7.9)	<0.001
CCI score 2	3881	(13.0)	302	(12.1)	
CCI score 3	4795	(16.1)	371	(14.8)	
CCI score 4	4605	(15.5)	409	(16.4)	
CCI score 5	3883	(13.1)	352	(14.1)	
CCI score 6	2818	(9.5)	315	(12.6)	
CCI score ≧7	6848	(23.0)	554	(22.2)	
**Medications**					
ACEI	10,015	(33.7)	1181	(47.2)	<0.001
ARB	8346	(28.1)	1176	(47.0)	<0.001
**Anticoagulation agents**	2974	(10.0)	421	(16.8)	<0.001
Gemfibrozil	1332	(4.5)	616	(24.6)	<0.001
Statin type	6528	(21.9)	1359	(54.4)	<0.001
Atorvastatin	2707	(9.1)	628	(25.1)	<0.001
Fluvastatin	1179	(4.0)	251	(10.0)	<0.001
Lovastatin	1121	(3.8)	227	(9.1)	<0.001
Pravastatin	580	(1.9)	116	(4.6)	<0.001
Rosuvastatin	843	(2.8)	220	(8.8)	<0.001
Simvastatin	1646	(5.5)	326	(13.0)	<0.001
**Hypoglycemic agents**	23,681	(79.6)	2348	(93.9)	<0.001
Metformin	17,940	(60.3)	2075	(83.0)	<0.001
Thiazolidinedione (TZD)	4763	(16.0)	822	(32.9)	<0.001
Sulfonylureas	21,939	(73.7)	2222	(88.9)	<0.001
Glinide	2414	(8.1)	403	(16.1)	<0.001
Acarbose	2968	(10.0)	582	(23.3)	<0.001
Insulin	112	(0.4)	4	(0.2)	0.083

Abbreviations: ACE-I: angiotensin-converting-enzyme inhibitor, ARB: angiotensin receptor blocker, IQR: interquartile range, and CCI: Charlson comorbidity index, SD: standard deviation.

**Table 2:** Hazard ratios of retinopathy based on the fenofibrate use status.

	Number of Subjects	Number of Retinopathy Events (%)	Year	Unadjusted HR (95%CI)	Adjusted HR (95%CI)
No Fenofibrate	29,753	8175 (27.48)	0–8	1.00	1.00
Fenofibrate	2500	492 (19.68)	0	0.17(0.13–0.21)	0.42(0.33–0.53)
			1	0.22(0.18–0.27)	0.47(0.39–0.58)
			2	0.30(0.25–0.35)	0.53(0.45–0.63)
			3	0.39(0.35–0.45)	0.60(0.53–0.68)
			4	0.53(0.48–0.58)	0.68(0.62–0.76)
			5	0.70(0.64–0.77)	0.77(0.70–0.85)
			6	0.93(0.83–1.03)	0.87(0.78–0.97)
			7	1.23(1.08–1.41)	0.99(0.86–1.14)
			8	1.64(1.39–1.95)	1.12(0.93–1.33)

HR: hazard ratio.

## Supplementary Materials

The following are available online at https://www.mdpi.com/1010-660X/56/8/385/s1: Table S1: Hazard ratio of retinopathy based on different cumulative duration treatment, Figure S1: Survival curves of retinopathy based on different cumulative duration treatment, Table S2: Hazard ratio of laser treatment based on cumulative duration treatment.

## Abbreviations

diabetic retinopathy	(DR)
proliferative diabetic retinopathy	(PDR)
diabetic macular edema	(DME)
vision threatening DR	(VTDR)
Fenofibrate Intervention and Event Lowering in Diabetes	(FIELD)
Collaborative Atorvastatin Diabetes Study	(CARDS)
angiotensin-converting enzyme inhibitors	(ACE-I)
angiotensin receptor blockers	(ARB)
thiazolidinedione	(TZD)
renin-angiotensin system	(RAS)
Bureau of National Health Insurance	(BNHI)
National Health Insurance Research Database	(NHIRD)
Defined Daily Dose	(DDD)
International Classification of Diseases, Ninth Revision, Clinical Modification	(ICD-9-CM)
Charlson comorbidity index	(CCI)
type 1 diabetes mellitus	(T1 DM)
type 2 diabetes mellitus	(T2 DM)
Hazard ratios	(HR)
proportional hazard	(PH)
interquartile range	(IQR)

## References

[1] Yau J.W., Rogers S.L., Kawasaki R., Lamoureux E.L., Kowalski J.W., Bek T., Chen S.J., Dekker J.M., Fletcher A., Grauslund J. (2012). Global prevalence and major risk factors of diabetic retinopathy. Diabetes Care.

[2] Wang F.H., Liang Y.B., Zhang F., Wang J.J., Wei W.B., Tao Q.S., Sun L.P., Friedman D.S., Wang N.L., Wong T.Y. (2009). Prevalence of diabetic retinopathy in rural China: The Handan Eye Study. Ophthalmology.

[3] Fong D.S., Aiello L.P., Ferris F.L., Klein R. (2004). Diabetic retinopathy. Diabetes Care.

[4] UK Prospective Diabetes Study (UKPDS) Group (1998). Intensive blood-glucose control with sulphonylureas or insulin compared with conventional treatment and risk of complications in patients with type 2 diabetes (UKPDS 33). Lancet.

[5] Nathan D.M., Genuth S., Lachin J., Cleary P., Crofford O., Davis M., Rand L., Siebert C., The Diabetes Control and Complications Trial Research Group (1993). The effect of intensive treatment of diabetes on the development and progression of long-term complications in insulin-dependent diabetes mellitus. N. Eng. J. Med..

[6] Chaturvedi N., Sjolie A.K., Stephenson J.M., Abrahamian H., Keipes M., Castellarin A., Rogulja-Pepeonik Z., Fuller J.H. (1998). Effect of lisinopril on progression of retinopathy in normotensive people with type 1 diabetes. The EUCLID Study Group. EURODIAB Controlled Trial of Lisinopril in Insulin-Dependent Diabetes Mellitus. Lancet.

[7] Keech A.C., Mitchell P., Summanen P.A., O’Day J., Davis T.M., Moffitt M.S., Taskinen M.R., Simes R.J., Tse D., Williamson E. (2007). Effect of fenofibrate on the need for laser treatment for diabetic retinopathy (FIELD study): A randomised controlled trial. Lancet.

[8] Chew E.Y., Ambrosius W.T., Davis M.D., Danis R.P., Gangaputra S., Greven C.M., Hubbard L., Esser B.A., Lovato J.F., Perdue L.H. (2010). Effects of medical therapies on retinopathy progression in type 2 diabetes. N. Eng. J. Med..

[9] Colhoun H.M., Betteridge D.J., Durrington P.N., Hitman G.A., Neil H.A., Livingstone S.J., Thomason M.J., Mackness M.I., Charlton-Menys V., Fuller J.H. (2004). Primary prevention of cardiovascular disease with atorvastatin in type 2 diabetes in the Collaborative Atorvastatin Diabetes Study (CARDS): Multicentre randomised placebo-controlled trial. Lancet.

[10] NHIRD Introduction to the National Health Insurance Research Database (NHIRD), Taiwan. http://nhird.nhri.org.tw/en/index.htm.

[11] Charlson M.E., Pompei P., Ales K.L., MacKenzie C.R. (1987). A new method of classifying prognostic comorbidity in longitudinal studies: Development and validation. J. Chronic Dis..

[12] Deyo R.A., Cherkin D.C., Ciol M.A. (1992). Adapting a clinical comorbidity index for use with ICD-9-CM administrative databases. J. Clin. Epidemiol..

[13] Beulens J.W., Patel A., Vingerling J.R., Cruickshank J.K., Hughes A.D., Stanton A., Lu J., McG Thom S.A., Grobbee D.E., Stolk R.P. (2009). Effects of blood pressure lowering and intensive glucose control on the incidence and progression of retinopathy in patients with type 2 diabetes mellitus: A randomised controlled trial. Diabetologia.

[14] Chaturvedi N., Porta M., Klein R., Orchard T., Fuller J., Parving H.H., Bilous R., Sjolie A.K. (2008). Effect of candesartan on prevention (DIRECT-Prevent 1) and progression (DIRECT-Protect 1) of retinopathy in type 1 diabetes: Randomised, placebo-controlled trials. Lancet.

[15] Villarroel M., Garcia-Ramirez M., Corraliza L., Hernandez C., Simo R. (2011). Fenofibric acid prevents retinal pigment epithelium disruption induced by interleukin-1beta by suppressing AMP-activated protein kinase (AMPK) activation. Diabetologia.

[16] Kim J., Ahn J.H., Kim J.H., Yu Y.S., Kim H.S., Ha J., Shinn S.H., Oh Y.S. (2007). Fenofibrate regulates retinal endothelial cell survival through the AMPK signal transduction pathway. Exp. Eye Res..

[17] Trudeau K., Roy S., Guo W., Hernandez C., Villarroel M., Simo R. (2011). Fenofibric acid reduces fibronectin and collagen type IV overexpression in human retinal pigment epithelial cells grown in conditions mimicking the diabetic milieu: Functional implications in retinal permeability. Investig. Ophthalmol. Vis. Sci..

[18] Chen Y., Hu Y., Lin M., Jenkins A.J., Keech A.C., Mott R., Lyons T.J., Ma J.X. (2013). Therapeutic effects of PPARalpha agonists on diabetic retinopathy in type 1 diabetes models. Diabetes.

[19] Leung H., Wang J.J., Rochtchina E., Wong T.Y., Klein R., Mitchell P. (2005). Dyslipidaemia and microvascular disease in the retina. Eye (Lond).

