# Supplementary material for: Association of Fenofibrate and Diabetic Retinopathy in Type 2 Diabetic Patients: A Population-Based Retrospective Cohort Study in Taiwan
Source: Medicina (Kaunas). 2020 Jul 31;56(8):385. doi: 10.3390/medicina56080385 (PMC7466234; doi:10.3390/medicina56080385)
Supplement: Supplementary file 1 [file medicina-56-00385-s001.pdf]

Table S1. Hazard ratio of retinopathy based on different cumulative duration treatment.

	**Number of subjects**	**Number of retinopathy events (%)**	**Year**	**Unadjusted HR (95%CI)**	**Adjusted HR (95%CI)**
Fenofibrate (<3M)	29753	8175 (27.48)	0-8	1	1
Fenofibrate (3M–1yr)	1698	360 (21.20)	0	0.23 (0.18–0.29)	0.52 (0.40–0.68)
			1	0.29 (0.23–0.36)	0.57 (0.46–0.71)
			2	0.37 (0.31–0.44)	0.63 (0.53–0.75)
			3	0.47 (0.41–0.54)	0.69 (0.60–0.80)
			4	0.60 (0.54–0.67)	0.76 (0.68–0.85)
			5	0.76 (0.69–0.85)	0.84 (0.75–0.94)
			6	0.97 (0.86–1.10)	0.92 (0.81–1.05)
			7	1.24 (1.05–1.45)	1.02 (0.86–1.20)
			8	1.58 (1.29–1.93)	1.12 (0.91–1.38)
Fenofibrate (1–2 yr)	531	91 (17.14)	0	0.09 (0.05–0.17)	0.28 (0.15–0.51)
			1	0.13 (0.08–0.22)	0.33 (0.20–0.55)
			2	0.19 (0.13–0.29)	0.40 (0.26–0.60)
			3	0.28 (0.20–0.39)	0.47 (0.34–0.65)
			4	0.41 (0.32–0.52)	0.57 (0.44–0.73)
			5	0.59 (0.48–0.73)	0.68 (0.55–0.84)
			6	0.85 (0.68–1.07)	0.81 (0.65–1.01)
			7	1.24 (0.93–1.64)	0.97 (0.73–1.29)
			8	1.79 (1.25–2.58)	1.16 (0.80–1.67)
Fenofibrate (>2yr)	271	41 (15.13)	0	0.02 (0.01–0.08)	0.07 (0.02–0.22)
			1	0.04 (0.02–0.12)	0.10 (0.03–0.27)
			2	0.08 (0.03–0.17)	0.14 (0.06–0.33)
			3	0.14 (0.07–0.26)	0.21 (0.11–0.40)
			4	0.24 (0.15–0.39)	0.31 (0.19–0.50)
			5	0.42 (0.29–0.61)	0.45 (0.31–0.65)
			6	0.75 (0.55–1.02)	0.66 (0.49–0.91)
			7	1.32 (0.92–1.90)	0.98 (0.67–1.42)
			8	2.33 (1.43–3.82)	1.44 (0.86–2.39)

HR: hazard ratio, CI: Confidence interval.

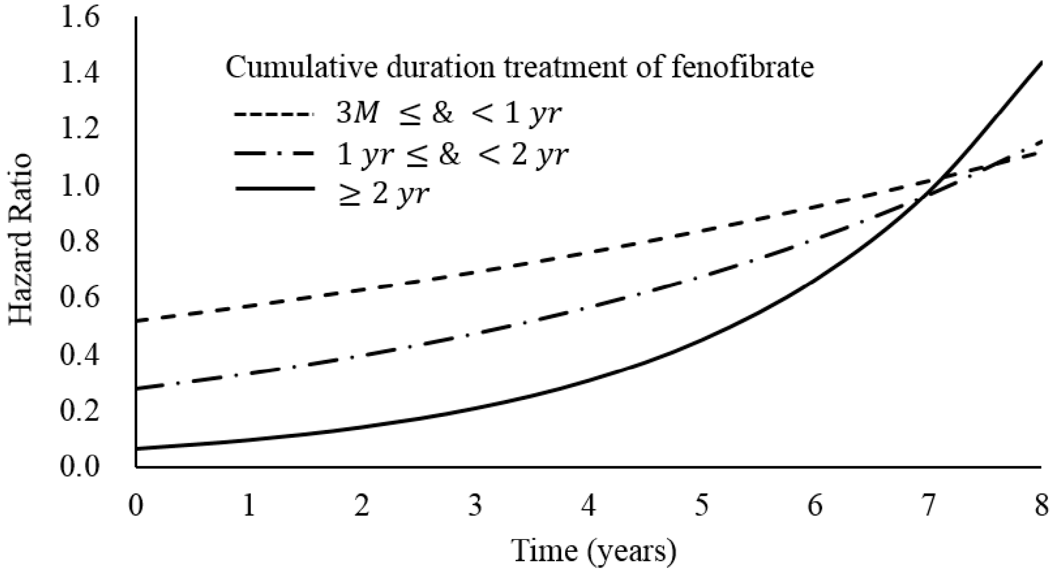

Figure S1. Survival curves of retinopathy based on different cumulative duration treatment.

Table S2. Hazard ratio of laser treatment based on cumulative duration treatment.

	**Number of subjects**	**Number of retinopathy events (%)**	**Year**	**Unadjusted HR (95% CI)**	**Adjusted HR (95% CI)**
No Fenofibrate	29713	1919 (6.46)	0–8	1.00	1.00
Fenofibrate	2540	124 (4.88)	0	0.15 (0.09–0.25)	0.31 (0.18–0.52)
			1	0.20 (0.13–0.31)	0.36 (0.23–0.56)
			2	0.27 (0.19–0.39)	0.42 (0.30–0.61)
			3	0.37 (0.28–0.48)	0.50 (0.38–0.65)
			4	0.50 (0.40–0.61)	0.58 (0.47–0.72)
			5	0.67 (0.56–0.81)	0.68 (0.56–0.82)
			6	0.90 (0.74–1.11)	0.80 (0.65–0.98)
			7	1.22 (0.94–1.57)	0.94 (0.72–1.22)
			8	1.64 (1.18–2.28)	1.10 (0.78–1.54)

HR: hazard ratio, CI: Confidence interval.
